# Endothelial glycocalyx degradation and disease severity in *Plasmodium vivax* and *Plasmodium knowlesi* malaria

**DOI:** 10.1038/s41598-021-88962-6

**Published:** 2021-05-07

**Authors:** Bridget E. Barber, Matthew J. Grigg, Kim A. Piera, Youwei Chen, Timothy William, J. Brice Weinberg, Tsin W. Yeo, Nicholas M. Anstey

**Affiliations:** 1 QIMR Berghofer Medical Research Institute, 300 Herston Rd, Herston, Brisbane, QLD 4006 Australia; 2 Menzies School of Health Research and Charles Darwin University, Darwin, Australia; 3 Infectious Diseases Society Sabah-Menzies School of Health Research Clinical Research Unit, Kota Kinabalu, Malaysia; 4 Duke University and V.A. Medical Centre, Durham, USA; 5 Clinical Research Centre, Queen Elizabeth Hospital, Kota Kinabalu, Malaysia; 6 Gleneagles Hospital, Kota Kinabalu, Malaysia; 7 Lee Kong Chian School of Medicine, Nanyang Technological University, Singapore, Singapore

**Keywords:** Biomarkers, Medical research, Pathogenesis

## Abstract

Degradation of the endothelial glycocalyx is associated with mortality in adult falciparum malaria. However, its role in the pathogenesis of non-falciparum malaria is unknown. In Malaysian patients with knowlesi (n = 200) and vivax (n = 61) malaria, and in healthy controls (n = 50), we measured glycocalyx breakdown products plasma syndecan-1 and urinary glycosaminoglycans, and evaluated correlations with biomarkers of disease severity. Urinary glycosaminoglycans were increased in patients with knowlesi and vivax malaria compared to healthy controls, and in knowlesi malaria were highest in those with severe disease. In knowlesi malaria, plasma syndecan-1 was also highest in those with severe disease, and correlated with markers of endothelial activation (angiopoietin-2, osteoprotegerin, ICAM-1), asymmetric dimethylarginine (ADMA) and impaired microvascular reactivity. Syndecan-1 also correlated with endothelial activation (ICAM-1, angiopoietin-2) and ADMA in vivax malaria. In knowlesi malaria increased syndecan-1 was associated with acute kidney injury, after controlling for age and parasitemia. In knowlesi malaria, the difference in median syndecan-1 between severe and non-severe disease was more marked in females than males. Endothelial glycocalyx degradation is increased in knowlesi and vivax malaria, and associated with disease severity and acute kidney injury in knowlesi malaria. Agents that inhibit glycocalyx breakdown may represent adjunctive therapeutics for severe non-falciparum malaria.

## Introduction

Malaria causes major morbidity and mortality worldwide. While *Plasmodium falciparum* is the major cause of malaria deaths globally, outside of Africa the non-falciparum species *Plasmodium vivax* and *Plasmodium knowlesi* cause significant disease burden, with both species able to cause severe and fatal disease^1–6^. *P. knowlesi* is a particular health problem in Malaysia, where this species now accounts for > 98% of all malaria cases^7^, and severe disease occurs in 6–9% of patients^8,9^. Complications include respiratory distress and acute kidney injury (AKI), with the latter particularly common in older adults^3, 9–11^. Given the long-term consequences of AKI^12^, adjunctive treatments of severe malaria are warranted to attenuate this complication. Development of adjunctive treatments requires improved understanding of the pathogenic mechanisms of severe disease.

As with falciparum malaria, severe knowlesi and vivax malaria are characterised by microvascular dysfunction, leading to impaired tissue perfusion and organ dysfunction^11,13, 14^. Contributing factors include intravascular haemolysis, impaired nitric-oxide (NO) bioavailability, and endothelial activation^11,13,14^. In falciparum malaria, degradation of the endothelial glycocalyx also occurs^15–18^, and in adults is associated with disease severity and mortality^15^. However, the role of the glycocalyx in knowlesi and vivax malaria has not been evaluated.

The endothelial glycocalyx is a gel-like layer that covers the endothelium, and maintains vascular haemostasis, tone and permeability (reviewed in^19,20^). It regulates endothelial cell NO release, prevents leakage of intravascular fluid and proteins, and attenuates leukocyte- and platelet-binding to endothelial adhesion receptors^19^. The glycocalyx is comprised of membrane-bound proteoglycans, glycosaminoglycan chains (GAGs), glycoproteins, and adherent plasma proteins (see^19,20^ for review, images and figures). The major glycosaminoglycans are heparan sulfate, chondroitin sulfate and the non-sulfated hyaluronan, while the proteoglycans include the syndecans and glycipans. When the glycocalyx degrades, a number of these components are shed into the plasma and/or urine and can be measured as biomarkers of glycocalyx breakdown. The best studied of these biomarkers include plasma syndecan-1, plasma heparin sulfate, urinary hyaluronan, and total urinary sulphated GAGs. Glycocalyx degradation has been increasingly recognized to play a key role in the pathogenesis of a number of other conditions, including sepsis^20^, dengue^21^ and most recently in severe COVID-19^22^. In sepsis, breakdown is mediated by glycocalyx “sheddases” such as heparanase, metalloproteinases, and hyaluronidase, activated by reactive oxygen species and proinflammatory cytokines^20^. Another activator of heparanase is the endothelial NO synthase (eNOS) inhibitor asymmetric dithemethylarginine (ADMA)^23^. ADMA is known to be elevated in falciparum malaria^24,25^ and sepsis^26^, and hence may contribute to glycocalyx breakdown in these conditions. While ADMA has not been found to be increased in vivax malaria^14^, the role of ADMA in knowlesi malaria has not been evaluated.

In this study, we measured concentrations of the glycocalyx breakdown products plasma syndecan-1 and urinary GAGs, and plasma concentrations of ADMA, in Malaysian adults with severe and non-severe knowlesi and vivax malaria, and in healthy controls. We assessed correlations between the glycocalyx breakdown products and endothelial activation (as measured by angiopoietin-2, osteoprotegerin [OPG] and intercellular adhesion molecule-1 [ICAM-1]), ADMA, microvascular function, and other biomarkers of disease severity. Plasma concentrations of the glycocalyx stabiliser sphingosine-1-phosphate (S-1-P) were also evaluated.

## Results

### Patients

A total of 261 patients with malaria were enrolled and had plasma and/or urine available for measurement of glycocalyx breakdown products, including 200 patients with knowlesi malaria (152 non-severe and 48 severe) and 61 with vivax malaria (52 non-severe, 9 severe). Plasma was available for 197 patients with knowlesi malaria (149 non-severe, 48 severe), and 58 patients with vivax malaria (50 non-severe, 8 severe). Urine was available for 126 patients with knowlesi malaria (85 non-severe, 41 severe) and 51 with vivax malaria (43 non-severe, 8 severe). Fifty healthy controls were enrolled; plasma syndecan-1 and S-1-P were measured in 20, and urinary GAGS were measured in 47. Clinical and pathophysiological data from these patients have been previously reported^10,11,13,14,27^. Baseline demographic, clinical and laboratory features are shown in Table 1. Patients were enrolled a median of 6 h (IQR 1.4–12.0 h) after commencement of antimalarial treatment.

**Table 1:** Baseline characteristics.

	*P. knowlesi*				*P. vivax*		
	Controls (N = 50*)	Non-severe (N = 152*)	Severe (N = 48*)	*P* value (Severe vs. NS)	Non-severe (N = 52*)	Severe (N = 9*)	*P* value (Severe vs. NS)
Age, years (median, range)	36 (17–59)	40 (13–94)	55 (20–81)	< 0.0001	24 (18–38)	39 (30–52)	0.146
Male sex, n (%)	15 (75)	121 (80)	35 (73)	0.329	39 (75)	9 (100)	0.184
Fever duration, days	NA	5 (3.5–7)	6 (3–7)	0.683	5 (3–7)	3 (3–4)	0.106
Time from malaria treatment to enrolment, hours	NA	5.8 (0–11.8)	6.5 (0–12.1)	0.134	4.9 (0–11.2)	9.7 (5.9–15.0)	0.141
Parasites/µL	NA	3782 (1012–13,868)	98,974 (24,034–164,304)	< 0.0001	4055 (1650–10,168)	10,242 (4387–19,520)	0.023
Platelet count, × 10^3^/uL	NA	51 (36–76)	31 (21–57)	< 0.001	70 (55–106)	29 (18–71)	0.020
Haemoglobin, g/dL, mean (SD)	NA	12.9 (1.5)	11.9 (2.1)	< 0.001	12.3 (2.1)	13.9 (1.2)	0.025
Creatinine, µmol/L	NA	95 (77–113)	144 (112–207)	< 0.0001	82 (68–99)	128 (94–136)	0.009
Bilirubin, µmol/L	NA	17 (13–25)	39 (24–88)	< 0.0001	17 (12–23) N = 49	26 (13–41)	0.138
Lactate, mmol/L	NA	1.2 (0.9–1.5) N = 134	1.5 (1.1–2.3)	< 0.001	1.2 (0.8–1.5) N = 47	1.3 (1.0–2.2)	0.200
ADMA, µM	0.52 (0.48–0.58) N = 40	0.49 (0.43–0.54) N = 106	0.60 (0.48–0.75)	< 0.0001	0.54 (0.44–0.62)	0.53 (0.50–0.54)	0.768
Angiopoietin-2, pg/ml	1,183 (875–1,597)	4296 (2943–6323)	10,072 (6,311–14,072)	< 0.0001	4598 (3196–6284)	8857 (6547–9743)	0.002
Osteoprotegerin, pg/ml	986 (625–1,463)	2087 (1605–3008)	4,795 (3,184–7,535) N = 46	< 0.0001	2463 (1172–5392)	4565 (2607–10,028)	0.059
ICAM-1, pg/ml	149 (123–167)	469 (363–621)	563 (430–703)	0.002	506 (413–639)	686 (682–832)	0.080
E-selectin, pg/ml	19 (13–25)	49 (36–66)	63 (50–90)	< 0.001	68 (42–92)	73 (65–115)	0.555
Microvascular reactivity, units/second	6.62 (5.43–7.25) N = 43	6.1 (5.3–6.9) N = 59	3.5 (2.8–5.3) N = 41	< 0.0001	6.9 (5.7–7.5) N = 32	5.4 (3.6–6.0) N = 8)	0.008
Urinary GAGs^#^ (g/mol creatinine)	0.06 (0.03–0.12)	1.32 (0.18–2.28) N = 85	2.39 (0.29v4.73) N = 41	0.016	1.12 (0.23–3.17) N = 43	1.46 (0.06–3.57) N = 8	0.613
Syndecan-1 (ng/ml)	177 (130–519) N = 20	154 (108–231) N = 149	283 (206–575)	< 0.0001	140 (109–205) N = 50	180 (154–326) N = 8	0.207
S-1-P (uM)	1.49 (1.1–2.4) N = 20	1.35 (1.01–1.86)	0.99 (0.58–1.6)	0.003	1.99 (1.29–3.17) N = 50	1.50 (0.97–2.81) N = 8	0.316

Numbers indicate median (IQR) unless otherwise indicated. * NA* not assessed, *NS* non-severe, *GAGs* glycosaminoglycans, *ADMA* asymmetric dimethylarginine, *ADMA* ICAM-1: intercellular adhesion molecule. *Unless otherwise indicated; ^#^GAGs above level of detection in 63% and 62% of patients with severe *P. knowlesi* and *P. vivax* malaria respectively, and in 69% and 67% of patients with non-severe *P. knowlesi* and *P. vivax* respectively. GAGs above level of detection in only 6% of controls.

As previously reported^10^, among the 48 patients with severe knowlesi malaria, WHO severity criteria included hyperparasitemia (n = 24, 50%), jaundice (n = 21, 44%), respiratory distress (n = 14, 29%), severe AKI (creatinine > 265 mmol/L; n = 11, 23%), shock (n = 11, 23%), metabolic acidosis (n = 4, 8%), severe anaemia (n = 5, 10%), and abnormal bleeding (n = 5, 8%). Using Kidney Disease: Improving Global Outcomes (KDIGO) criteria to define AKI, and the Modification of Diet in Renal Disease [MDRD] equation to estimate baseline creatinine, AKI was present on or during admission in 31 (65%) patients with severe malaria, and 14 (9%) patients with non-severe malaria. Of the 9 patients with severe vivax malaria, severity criteria included shock (n = 6, 67%), jaundice (n = 2, 22%), respiratory distress (n = 1, 11%), metabolic acidosis (n = 1, 11%), abnormal bleeding (n = 1, 11%), and convulsions (n = 1, 11%). AKI by KDIGO criteria occurred in 3 (33%) patients with severe vivax malaria, and 3 (6%) with non-severe vivax malaria.

### Glycocalyx breakdown products and disease severity in knowlesi and vivax malaria

**Figure 1:**
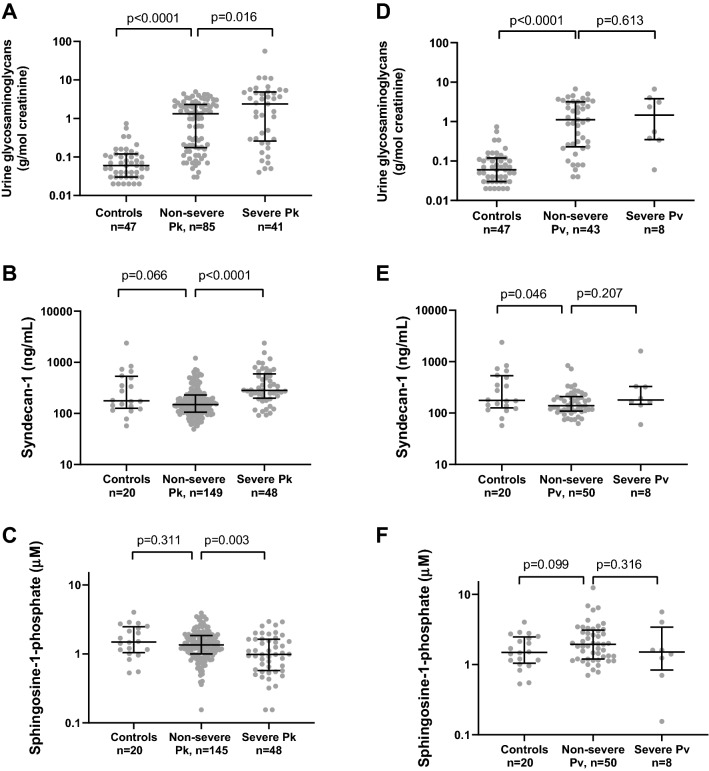
Glycocalyx breakdown products, and sphingosine-1-phosphate, in patients with knowlesi (**A**–**C**) and vivax (**D**–**F**) malaria, and in healthy controls. Errors bars indicate median and inter-quartile range. For comparisons between severe knowlesi malaria and controls, the *P* values are < 0.0001, 0.185, and 0.013 for panels (**A**), (**B**) and (**C**), respectively. For comparisons between severe knowlesi malaria and controls, the *P* values are < 0.001, 0.899, and 0.980, for panels (**D**), (**E**) and (**F**), respectively.

In patients with knowlesi malaria, urinary concentrations of GAGs were higher in those with severe compared to non-severe disease (median 2.36 [IQR 0.29–4.73] vs. 1.32 [IQR 0.18–2.28] g/mol creatinine, *p* = 0.016), and were higher in both disease groups compared to controls (0.06 [IQR 0.03–0.12] g/mol creatinine, *p* < 0.0001 for both comparisons; Table 1 and Fig. 1). Similarly, plasma concentrations of syndecan-1 were higher in patients with severe disease compared to non-severe disease (median 283 [IQR 206–575] vs. 154 [IQR 106–231] ng/ml, *p* < 0.0001). Surprisingly, concentrations of syndecan-1 were lower in those with non-severe disease compared to healthy controls (median 177 [IQR 130–519 ng/ml]), although this was not statistically significant. Concentrations of the glycocalyx stabilizer S-1-P were lower in patients with severe compared to non-severe knowlesi malaria (median 0.19 [IQR 0.58–1.63] vs. 1.35 [IQR 1.01–1.86] µM, *p* = 0.003); however, again there was no significant difference between those with non-severe disease and healthy controls (median 1.49 [IQR 1.08–2.44] µM).

In vivax malaria, as with knowlesi malaria, the median urinary GAGs was higher in patients with severe (1.46 [IQR 0.36–3.57] g/mol creatinine) and non-severe (1.12 [IQR 0.23–3.17] g/mol creatinine) disease compared to healthy controls (0.06 [IQR 0.03–0.12] g/mol creatinine, *p* < 0.001 for both comparisons; Table 1 and Fig. 1). However, there was no difference observed between patients with severe and non-severe vivax malaria. Plasma concentrations of syndecan-1 were again unexpectedly reduced in patients with non-severe disease compared to healthy controls (median 140 [IQR 109–205] vs. 177 [IQR 130–519 ng/ml *p* = 0.046). Concentrations of plasma syndecan-1 were higher in those with severe (median 180 [IQR 154–326] ng/ml) compared to non-severe disease, although numbers were small and this was not significant. In patients with vivax malaria there was no significant difference in concentrations of S-1-P between disease groups, or between either disease group and controls (Table 1 and Fig. 1).

Plasma concentrations of syndecan-1 were correlated with urinary GAGs in knowlesi malaria (*r* = 0.21, *p* = 0.019), although this did not remain significant after controlling for parasitemia. There was no correlation between plasma syndecan-1 and urinary GAGs in vivax malaria. There was also no correlation between S-1-P and either plasma syndecan-1 or urinary GAGs, in either knowlesi or vivax malaria.

Plasma concentrations of syndecan-1 were correlated with duration of fever in knowlesi malaria (*r* = 0.30, *p* < 0.0001) and in vivax malaria (*r* = 0.49, *p* < 0.001); in both species this remained significant after controlling for parasitemia (knowlesi malaria: *p* < 0.001; vivax malaria: *p* = 0.020). No such correlation occurred with urinary GAGs, in either knowlesi or vivax malaria. There was no difference in syndecan-1 or urinary GAGs in malaria patients enrolled before, or after, commencing antimalarial treatment.

### Asymmetric dimethylarginine and disease severity in knowlesi malaria

In patients with knowlesi malaria, the median concentration of ADMA was higher in patients with severe compared to non-severe disease (0.60 [IQR 0.48–0.75] vs. 0.49 [IQR 0.43–0.54] µM, *p* < 0.0001), and higher in those with severe disease compared to controls (0.52 [IQR 0.48–0.58], *p* = 0.014). As with syndecan-1, concentrations of ADMA were unexpectedly lower in those with non-severe disease compared to controls (*p* = 0.007). The concentrations of ADMA in patients with severe knowlesi malaria were comparable to those of patients with severe falciparum malaria, enrolled in the same study cohort (median 0.57 [IQR 0.52–0.65] µM, N = 22^24^).

### Glycocalyx breakdown products and biomarkers of severity in knowlesi and vivax malaria

In patients with knowlesi malaria, biomarkers of severity were more closely correlated with syndecan-1 than with urinary GAGs (Table 2). After applying Bonferonni’s correction for multiple comparisons (level of statistical significance, *p* < 0.0045) and adjusting for parasitemia, syndecan-1 was associated with ADMA (*r* = 0.33, *p* < 0.0001), creatinine (*r* = 0.46, *p* < 0.0001), lactate (*r* = 0.25, *p* < 0.001), angiopoietin-2 (*r* = 0.48, *p* < 0.0001), intercellular adhesion molecule 1 (ICAM-1; *r* = 0.30, *p* < 0.0001), osteoprotegerin (OPG; *r* = 0.25, *p* < 0.001), and inversely with microvascular reactivity (*r* = − 0.36, *p* < 0.001). Syndecan-1 was also inversely correlated with albumin (*r* = − 0.41, *p* < 0.0001), sodium (*r* = − 0.24, *p* < 0.001), bicarbonate (*r* = − 0.31, *p* < 0.0001), and chloride (*r* = − 0.21, *p* = 0.004).

**Table 2:** Correlation coefficients between glycocalyx breakdown products and markers of severity in knowlesi malaria.

Biomarker	Univariate analysis		Controlling for parasitemia	
	Correlation	*P* value	Correlation	*P* value
**Urinary GAGs (n = 126)**				
Parasitemia	0.21	0.017		
Platelet count	− 0.13	0.145		
Haemoglobin	− 0.02	0.819		
Creatinine	0.06	0.513		
Lactate	0.06	0.524		
ADMA	0.17	0.055		
Angiopoietin-2	0.18	0.048	0.06	0.501
Osteoprotegerin	0.23	0.010	0.13	0.145
ICAM-1	0.28	0.001	0.24	0.008
E-selectin	0.26	0.004	0.18	0.049
Microvascular reactivity	− 0.20	0.061		
**Syndecan-1 (n = 197)**				
Parasitemia	0.13	0.075		
Platelet count	− 0.26	< 0.001	− 0.23	0.001
Haemoglobin	− 0.23	0.001	− 0.27	< 0.001
Creatinine	0.50	< 0.0001	0.46	< 0.0001
Lactate	0.23	0.002	0.25	< 0.001
ADMA	0.27	0.001	0.33	< 0.0001
Angiopoietin-2	0.46	< 0.0001	0.48	< 0.0001
Osteoprotegerin	0.27	< 0.001	0.25	< 0.001
ICAM-1	0.32	< 0.0001	0.30	< 0.0001
E-selectin	0.22	0.002	0.20	0.006
Microvascular reactivity	− 0.41	< 0.0001	− 0.36	< 0.001

After applying Bonferonni’s correction for the 11 variables evaluated in the correlation analyses above, correlations were considered statistically significant if *p* < 0.0045.

*GAGs* glycosaminoglycans, *ADMA* asymmetric dimethylarginine, *ICAM-1* intercellular adhesion molecule-1.

In patients with knowlesi malaria, in a logistic regression model controlling for age and parasitemia, log-transformed plasma syndecan-1 was associated with severe disease (OR 3.72 [95% CI 2.01–6.89], *p* < 0.0001); this remained significant after controlling for the other biomarkers of severity listed above. After controlling for age and parasitemia, log-transformed syndecan-1 was also associated with acute kidney injury (OR 3.25, *p* < 0.0001), again remaining significant after controlling for the other biomarkers listed above.

In vivax malaria (Table 3), urinary GAGs correlated with angiopoietin-2 (*r* = 0.41, *p* = 0.003), ADMA (*r* = 0.40, *p* = 0.004), and ICAM-1 (*r* = 0.54, *p* < 0.0001), with the correlation with ADMA and ICAM-1 remaining significant after adjusting for parasitemia (*p* = 0.0001). After adjusting for parasitemia, plasma syndecan-1 also correlated with ADMA (*r* = 0.45, *p* < 0.001), angiopoietin-2 (*r* = 0.44, *p* = 0.002), and ICAM-1 (*r* = 0.53, *p* < 0.0001). In vivax malaria, neither urinary GAGs nor plasma syndecan-1 were independently associated with risk for severe disease, or AKI.

**Table 3:** Partial correlation coefficients between glycocalyx breakdown products and markers of severity in vivax malaria.

Biomarker	Univariate analysis		Controlling for parasitemia	
	Correlation	*P* value	Correlation	*P* value
**Urinary GAGs (n = 51)**				
Parasitemia	0.22	0.128		
Platelet count	− 0.37	0.008	− 0.33	0.018
Haemoglobin	0.24	0.090		
Creatinine	− 0.19	0.175		
Lactate	− 0.01	0.960		
ADMA	0.40	0.004	0.42	0.002
Angiopoietin-2	0.41	0.003	0.24	0.096
Osteoprotegerin	0.07	0.634		
ICAM-1	0.54	< 0.0001	0.52	< 0.001
E-selectin	0.35	0.012	0.25	0.077
Microvascular reactivity	0.15	0.384		
**Syndecan-1 (n = 58)**				
Parasitemia	− 0.03	0.811		
Platelet count	− 0.38	0.003	− 0.50	< 0.001
Haemoglobin	− 0.15	0.257		
Creatinine	0.14	0.285		
Lactate	0.25	0.069		
ADMA	0.49	< 0.001	0.45	< 0.001
Angiopoietin-2	0.37	0.004	0.44	< 0.001
Osteoprotegerin	− 0.01	0.937		
ICAM-1	0.44	< 0.001	0.53	< 0.0001
E-selectin	0.03	0.822		
Microvascular reactivity	− 0.14	0.412		

After applying Bonferonni’s correction for the 11 variables evaluated in the correlation analyses above, correlations were considered statistically significant if p < 0.0045.

*GAGs* glycosaminoglycans, *ADMA* asymmetric dimethylarginine, *ICAM-1* intercellular adhesion molecule-1.

### Glycocalyx breakdown products and S-1-P, and correlations with haemoglobin and platelets

Red blood cells (RBCs) and platelets are both known to produce the glycocalyx stabiliser S-1-P^28,29^, and therefore anaemia and/or thrombocytopenia could potentially lead to reduced S-1-P with consequent glycocalyx breakdown. In patients with knowlesi malaria, syndecan-1 was inversely correlated with haemoglobin on enrolment (*r* = − 0.23, *p* = 0.001; Table 2), remaining significant after controlling for parasitemia and fever duration (*p* < 0.0001). However, there was no correlation between haemoglobin and S-1-P (*r* = 0.09, *p* = 0.216). In knowlesi malaria, syndecan-1 was also inversely correlated with platelet count on enrolment (*r* = − 0.23, *p* = 0.001), again remaining significant after controlling for parasitemia (*r* = − 0.23, *p* = 0.001). Platelet count was also correlated with S-1-P (*r* = 0.36, *p* < 0.0001), remaining significant after controlling for parasitemia (*r* = 0.28, *p* = 0.001).

As with *P. knowlesi*, in patients with vivax malaria syndecan-1 was inversely correlated with platelet count on enrolment, remaining significant after controlling for parasitemia (*r* = − 0.50, *p* = 0.001). Platelet count on enrolment was also correlated with S-1-P (*r* = 0.29, *p* = 0.026), remaining significant after controlling for parasitemia (*r* = − 0.33, *p* = 0.013). In vivax malaria there was no correlation between either syndecan-1 or S-1-P with haemoglobin.

### Correlations with age and sex

In patients with knowlesi malaria, median plasma syndecan-1 was higher in males compared to females (187 [IQR 123–307] vs. 133 [IQR 95–264] ng/ml, *p* = 0.014). Interestingly however, this difference occurred only in those with non-severe disease (168 [IQR 115–242] ng/ml in males vs. 110 [82–154] ng/ml in females, *p* < 0.001); in patients with severe knowlesi malaria, median plasma syndecan-1 was as high in females (281 [IQR 234–610] ng/ml) as it was in males (286 [IQR 192–541] ng/ml). Thus, in females, median syndecan-1 was 2.6-fold higher in severe compared to non-severe knowlesi malaria, while in males, the median was only 1.7-fold higher. Median syndecan-1 was also higher in healthy male controls (180 [IQR 143–663] ng/ml, n = 15) compared to healthy female controls (122 [IQR 115–350] ng/ml, n = 5), although numbers were small and this was not statistically significant. There were no significant sex differences in urinary GAGs in patients with knowlesi malaria. Median plasma S-1-P was lower in males compared to females (1.23 [IQR 0.89–1.82] vs. 1.63 [IQR 1.01– 1.91] µM, *p* = 0.05), with this difference observed in both severity groups.

In patients with vivax malaria there was a trend towards higher median plasma syndecan-1 in males compared to females (158 [IQR 118–229] vs. 109 [77–181] ng/ml, *p* = 0.068), and lower S-1-P (1.82 [IQR 1.24–2.64] vs. 3.2 [IQR 1.44–3.85] µM, *p* = 0.07). There were no significant sex differences in urinary GAGs in patients with vivax malaria.

There was a weak correlation between age and syndecan-1 in patients with knowlesi malaria (*r* = 0.16, *p* = 0.027); however, this did not remain significant after controlling for parasitemia. There was no correlation between age and syndecan-1 in patients with vivax malaria. There were also no significant correlations between age and S-1-P, in malaria patients or in controls.

## Discussion

In this study, we demonstrate that urinary GAGs, markers of glycocalyx breakdown, are increased in knowlesi and vivax malaria, and in knowlesi malaria are increased in proportion to disease severity. In knowlesi malaria the plasma marker of glycocalyx degradation, syndecan-1, was also elevated in severe compared to non-severe disease, and was independently correlated with nearly all biomarkers of severity evaluated, including endothelial activation, microvascular dysfunction, creatinine, and lactate. In knowlesi malaria, in logistic regression models syndecan-1 was also independently associated with both severe disease and AKI. Taken together these findings suggest that glycocalyx breakdown is likely a key contributor to disease pathogenesis in knowlesi malaria.

The finding that urinary GAGs, but not syndecan-1, were increased in non-severe knowlesi malaria, but that syndecan-1 correlated more strongly with biomarkers of severity, suggests that shedding of negatively charged GAG sidechains, which comprise the most superficial layer of the intraluminal glycocalyx, may occur earlier in the disease process. In contrast, shedding of syndecan-1, a deeper core glycocalyx protein attached to endothelial cells through a transmembrane domain, may occur later in disease, reflecting greater glycocalyx damage with greater functional impairment. The correlation of syndecan-1, but not urinary GAGs, with fever duration, is consistent with this hypothesis.

Consistent with previous reports in falciparum malaria^15,16^, we found that in both knowlesi and vivax malaria, syndecan-1 was associated with the endothelial activation marker angiopoietin-2, likely reflecting the known role of angiopoietin-2 in mediating glycocalyx breakdown^30^. Syndecan-1 was also associated with the adhesion molecule ICAM-1 in both knowlesi and vivax malaria. ICAM-1 is contained within the glycocalyx, and the correlation between syndecan-1 and ICAM-1 likely reflects shedding of these adhesion molecules. Glycocalyx degradation may also expose adhesion molecules; while this has been shown in vitro to increase endothelial cytoadherence of *P. falciparum*-infected red cells^31,32^, the consequences in knowlesi and vivax malaria are less certain, given the apparent paucity of endothelial cytoadherence with these species^33,34^.

We also found that syndecan-1 was associated with OPG, a soluble decoy receptor of receptor activator of NF-kB (RANK) ligand. In addition to the ability of OPG to bind to syndecan-1^35^, OPG has also been shown to inhibit RANK/RANK ligand-activation of eNOS, and may therefore contribute to glycocalyx breakdown by reducing eNOS-mediated inhibition of heparanase expression^23^. Increased heparanase expression may lead to glycocalyx degradation not just by the cleavage of heparan sulfate chains from core proteoglycans^36^, but also by activating metalloproteinases^37^, which cleave proteoglycans such as syndecan-1 directly from endothelial cells^38^.

Heparanase expression may also be induced by the eNOS inhibitor ADMA^23^. Although ADMA is known to be elevated in severe falciparum malaria, an association with glycocalyx breakdown has not previously been evaluated. In this study we found that ADMA was increased in severe knowlesi malaria, and was associated with both syndecan-1 and urinary GAGs. The findings in our study suggest that in addition to the role of angiopoietin-2 in mediating glycocalxy breakdown in malaria, agents that increase heparanase expression such as ADMA and OPG may also contribute. Importantly, ADMA and OPG are both increased in other conditions associated with glycocalyx breakdown, such as sepsis^26, 39^, and may represent common mechanisms of glycocalyx degradation. It is notable that in vivax malaria, an increase in ADMA is not observed^14^, and it is possible that this may account for the lack of any observed increase in plasma syndecan-1 in these patients.

Our finding that glycocalyx breakdown was an independent risk factor for AKI in knowlesi malaria is consistent with the key role that the glycocalyx in known to play in kidney function, with the glomerular glycocalyx regulating exchange of plasma proteins across glomerular capillaries^40^. Mouse models have demonstrated that glycocalyx degradation increases glomerular permeability to albumin, leading to albuminuria^41^. We have found that microalbuminuria is common in AKI complicating knowlesi malaria (unpublished), further suggesting that glycocalyx breakdown may be a key mechanism of malaria-associated AKI. Albumin is also able to stabilise glycocalyx^42^, and hence hypoalbuminemia may in itself exacerbate glycocalyx degradation.

A notable finding is our study was the inverse correlation in knowlesi malaria between syndecan-1 and haemoglobin, with this correlation remaining significant after controlling for parasitemia and fever duration. RBCs are known to be major producers of the glycocalyx stabiliser S-1-P^28^; however, we did not find a correlation between S-1-P and haemoglobin, and therefore reduced RBC production of S-1-P does not seem a likely explanation for the increased glycocalyx breakdown and elevated levels of syndecan-1. Alternatively, shedding of glycocalyx components from RBCs themselves may lead to increased plasma syndecan-1, given that RBCs, like endothelial cells, are known to contain a glycocalyx^43^. Shedding of glycocalyx from RBCs could further exacerbate anaemia, due to exposure of phosphatidylserine or other receptors enhancing eryptosis and/or phagocytosis.

We also found independent inverse correlations between platelets and both syndecan-1 and GAGs, in both vivax and knowlesi malaria. As with RBCs, platelets have been shown to produce S-1-P^29^, and in our study S-1-P was independently associated with platelet count. Thus, it is possible that reduced platelet-derived S-1-P could potentiate glycocalyx breakdown in malaria. Alternatively, glycocalyx breakdown may contribute directly to thrombocytopenia by mediating platelet adhesion and aggregation^44,45^.

We observed sex differences in glycocalyx degradation products. In patients with knowlesi malaria, syndecan-1 was higher and S-1-P lower, in males compared to females. This difference however was only observed in those with non-severe disease, and is consistent with a previous study in acute coronary syndrome demonstrating that males shed more glycocalyx than females^46^, and another study demonstrating that males have a thicker glycocalyx than females^47^. Consistent with this, we also found a trend towards higher syndecan-1 in male controls compared to female controls. Interestingly we found that in severe knowlesi malaria, syndecan-1 was as high in females compared to males, suggesting that severe disease in females may be associated with a greater proportionate loss of glycocalyx. In knowlesi malaria female sex is an independent risk factor for death^3^. While the cause of this increased risk is not well understood, it is possible that a thinner glycocalyx in females and a greater proportionate loss may contribute. Sex differences in disease severity and outcomes are observed in many acute infections^48, 49^; whether differences in glycocalyx degradation contribute warrants further evaluation.

Our study had some limitations. First, the small number of cases of severe vivax malaria limited our ability to examine relationships between markers of glycocalyx degradation and disease severity in vivax malaria. However, with larger numbers of cases of severe knowlesi malaria clear associations with disease severity were found. Second, the median age of the control group was higher than that of the patients with vivax malaria; however, given that we did not find significant correlations between age and markers of glycocalyx breakdown, this is unlikely to have affected our results. Third, our study did not have a non-malaria fever comparison group; however, it is well documented that glycocalyx degradation is not specific to malaria fevers, with degradation also found in a number of other conditions including sepsis^20^, dengue^21^ and COVID-19^22^. Finally, although we found associations between markers of glycocalyx degradation and biomarkers of disease severity, the nature of our study does not provide definitive evidence of causation.

In conclusion, we found that glycocalyx breakdown occurs in patients with knowlesi and vivax malaria, and is associated with disease severity and AKI in knowlesi malaria. Plasma syndecan-1 appeared to be a more clinically relevant biomarker than urinary GAGs, and may reflect more severe glycocalyx damage, occurring later in disease. Several agents have been demonstrated to stabilise endothelial glycocalyx, and may be potential agents for adjunctive treatment of severe non-falciparum malaria.

## Methods

### Ethics statement

The study was approved by the Ethics Committees of the Malaysian Ministry of Health and Menzies School of Health Research. Informed written consent was provided by all adults, and by the parent or guardian of any participant aged < 18 years.

### Study site and patients

Patients were enrolled as part of a prospective observational study of all malaria patients admitted to Queen Elizabeth Hospital, an adult tertiary-referral hospital in Sabah, Malaysia^11,27^. For the current study, patients enrolled between September 2010 and December 2012 were included if they had PCR-confirmed *P. knowlesi* or *P. vivax* monoinfection, were non-pregnant, ≥ 12 years old, had no major comorbidities or concurrent illness, were within 18 h of commencing antimalarial treatment, and had stored frozen urine and/or plasma available for measurement of glycocalyx breakdown products. Severe malaria was defined according to modified WHO criteria^50^, as previously described^11^. Renal function was further assessed using the Kidney Disease: Improving Global Outcomes (KDIGO) criteria for AKI^51^. Healthy controls were visitors or relatives of malaria patients, with no history of fever in the past 48 h and with blood film negative for malaria parasites.

Standardized history and physical examination were documented. Haematology, biochemistry, and lactate (by bedside blood analysis; iSTAT system) were obtained on enrolment. Parasite counts were determined by microscopy, and parasite species identified by PCR^52,53^. Patients with severe disease were treated with intravenous artesunate, while those with non-severe disease received oral artemisinin combination treatment (and 14 days of primaquine for those with vivax malaria), as previously described^27^.

### Laboratory assays and measurements of microvascular function

Venous blood collected on enrolment in lithium heparin and citrate tubes was centrifuged within 30 min of collection and plasma stored at – 70 °C. Urine was also collected on enrolment and stored within 30 min at – 70 °C. ELISA was used to measure heparanized plasma concentrations of syndecan-1 (Abcam); sphingosine-1-phosphate (S-1-P; Echelon Biosciences); angiopoietin-2, ICAM-1 and E-selectin (Quantikine R&D Systems) and osteoprotegerin (DuoSet R&D Systems). Cell free haemoglobin was measured on citrated platelet-free plasma by ELISA (Bethyl Laboratories). Heparanized plasma concentrations of the endothelial nitric oxide synthase (eNOS)-inhibitor asymmetric dimethylarginine (ADMA) were measured by HPLC^54^. Total urinary sulfated GAGs were measured on a subset by the dimethylmethylene blue (DMMB) colorimetric assay as previously described^15,55^. Urinary creatinine was measured by the alkaline picrate method, and the urine GAG concentrations were normalised to creatinine levels. All samples were diluted as per manufacturers’ recommendations but were adjusted if the resulting OD was outside the standard curve. Microvascular function was assessed using Near Infrared Spectroscopy (InSpectra 650, Hutchinson, MN), as previously described^56^. These data have been previously reported^11^, and are included here to assess correlation with glycocalyx degradation.

All methods were carried out in accordance with relevant guidelines and regulations.

### Statistics

Statistical analysis was performed with STATA software (v15). Student’s T-test or Wilcoxon-Mann–Whitney tests were used for two-group comparisons, depending on distribution. Categorical variables were compared using χ^2^ or Fisher’s exact tests. Associations between continuous variables were assessed using Spearman’s correlation coefficient. Partial correlation was used to evaluate associations between continuous variables after adjusting for parasitemia. For this analysis, non-normally distributed variables were log-transformed to a normal distribution. Since correlations between glycocalyx breakdown products (urinary glycosaminoglycans and syndecan-1) and 11 variables were assessed, Bonferroni’s correction was used to adjust for multiple correlations, with correlations considered statistically significant if *p* < 0.0045 (*p* = 0.05/11).

Backward stepwise regression was used to evaluate factors associated with severe malaria and AKI, with variables removed at a significance level of > 0.05; for this analysis, patients with hyperparasitemia as a sole severity criterion were reclassified as having non-severe malaria.

## Data Availability

The datasets generated during and/or analysed during the current study are available from the corresponding author on reasonable request and with an appropriate data sharing agreement.
